# Retrospective Study Reveals Decades of PCV4 Circulation in Spain

**DOI:** 10.1155/tbed/9326570

**Published:** 2025-07-22

**Authors:** Rocío Holgado-Martín, Luis Gómez, David Risco, Remigio Martínez, Alfredo García-Sánchez, Matteo Legnardi, Giovanni Franzo

**Affiliations:** ^1^Department of Animal Medicine, Area of Anatomy and Pathological Anatomy, University of Extremadura 10003, Cáceres, Spain; ^2^Department of Animal Health, Animal Health and Zoonosis Research Group (GISAZ), UIC Zoonosis and Emerging Diseases (ENZOEM), University of Córdoba 14014, Córdoba, Spain; ^3^Animal production, Scientific and Technological Research Center of Extremadura, (CICYTEX), km 372 06187, Badajoz, Guadajira, Spain; ^4^Department of Animal Medicine, Production and Health (MAPS), Padua University 35020, Legnaro, Italy

**Keywords:** PCV4, phylodynamic, phylogeographic, retrospective, Spain

## Abstract

Porcine circovirus 4 (PCV4) was first identified in China in 2019 and retrospectively traced back to 2008. However, unlike other circoviruses, its distribution appeared to be largely confined to Asian countries until recent reports from Spain and the United States. This study aims to enhance knowledge of the past and present circulation of PCV4 in non-Asian countries, particularly in Spain and in wild boars, where it was previously detected at a significant prevalence. By genetically characterizing the strains, the contextualization within both national and international epidemiological frameworks was attempted. A total of 302 lymph node samples were tested, with 62 testing positives by quantitative real-time polymerase chain reaction (qPCR), predominantly from the 2011 to 2015 period, although five positives were detected in the 2022–2024. Complete open reading frame 2 (ORF2) sequences were obtained from 10 strains. Phylogenetic analysis revealed two major clusters: one comprising only Chinese sequences and another containing strains from multiple continents, including Spain and the United States. The Spanish strains formed a distinct monophyletic clade whose introduction in the country was estimated through phylodynamic analyses around 2000, suggesting long-term undetected circulation. Within Spain, a progressive geographical spread and strain exchange between wild boars and outdoor reared domestic pigs could be inferred. On the other hand, the lack of detection in intensively raised pigs, at least in Europe, remains unclear. The present findings extend the current knowledge of PCV4 history outside Asia and challenge the paradigm of a recent international spreading. Recognizing the current uncertainties in PCV4's international distribution and epidemiology, further efforts are needed, including the use of archived samples in diagnostic activities as well as the sharing of negative results.

## 1. Introduction

Porcine circovirus 4 (PCV4) is an emerging viral species within the *Circoviridae* family, characterized by its small, circular, and single-stranded DNA genome. The discovery of PCV4 follows that of PCV1, PCV2, and PCV3. First identified in 2019 in China, PCV4 has been detected in both domestic pigs and wild boars, raising concerns regarding its potential impact on animal health, also based on the potentially shared behavior with other PCV proven to be pathogenic, that is, PCV2 and PCV3 [1–3]. However, there are currently no strong evidences of PCV4 pathogenic role in field conditions. Moreover, other peculiarities differentiate it from previously known PCVs. The geographic distribution of other PCVs is considered practically ubiquitous [4, 5], while PCV4 has been detected mainly in Asian countries, with just sporadic occurrences in other areas, limited to Spain and the USA [6–12], despite studies indicating its existence since at least 2008 in China and 2011 in Spain [7, 13, 14].

The genome of PCV4 contains 1770 bases and a palindrome stem-loop structure, with the conserved nona-nucleotide (CAGTATTAC) located within the intergenic region between two major open reading frames (ORFs), that are also present in the other types of PCVs [1]. ORF1 encodes the replicase protein (*Rep*) and ORF2 encodes the capsid protein (*Cap*). Alignment of sequences revealed that PCV4 has low identities with PCV1, PCV2, and PCV3 [1], while a closer relationship with mink circovirus (MiCV) has been reported [15]. Furthermore, this similarity to MiCVs suggests potential cross-species transmission events [16]. In fact, PCV4 DNA has also been detected in nonporcine species, such as dairy cows and dogs [17, 18].

PCV2 and PCV3 are usually associated with postweaning multisystemic wasting syndrome (PMWS) and porcine dermatitis and nephropathy syndrome (PDNS). Comparable signs and lesions have also been reported in the presence of PCV4 infection. However, the clinical signs caused by PCV4 infection are still not established as it has been detected in animals showing several generic symptoms, such as respiratory symptoms, neurological symptoms, diarrhea, enteritis, encephalitis, and skin disease [4, 16, 19, 20], and moreover, has been detected in asymptomatic animals [1]. The absence of a clear pathogenesis highlights the need for further epidemiological investigations.

The wild boar (*Sus scrofa*) serves as a natural reservoir for numerous pathogens, including PCVs, due to its wide distribution, genetic diversity, and frequent interactions with domestic species [21]. The role of wild boars in the epidemiology of PCV4 has demonstrated to be important in Spain, both for virus maintenance and because of connection with domestic animals. In fact, pigs reared outdoors (Iberian pig breed) in the same areas where high wild boar PCV4 circulation was proven were also found PCV4 positive [7]. The presence of this virus in Spain and the substantial wild boar population presents a unique opportunity to investigate PCV4 historic circulation and evolution [22].

The aim of this study was to genetically characterize the PCV4 strains obtained from wild boar samples collected over time across various provinces in Spain. By analyzing the genetic diversity and evolutionary relationships of PCV4 strains, we seek to provide insights into the virus's molecular origin, epidemiology, evolution, and its potential interactions with other strains throughout the world, contributing to PCV4 historical dynamics understanding.

## 2. Material and Methods

### 2.1. Experimental Design

**Figure 1:**
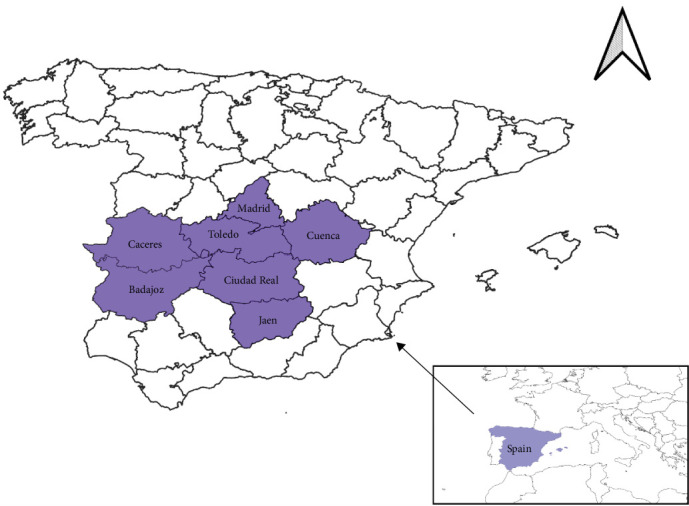
Provinces of the sampled area of mid-western Spain.

Archive samples of lymph nodes from wild boars were included in the study to retrospectively analyze PCV4 presence. All samples were frozen at −20°C after collection until use. Prior to DNA extraction, the samples were thawed and homogenized using molecular biology-grade water and a stomacher, and the extraction was performed from this homogenate. Two groups of samples were available, originating from different sampling campaigns, performed from 2011 to 2015 and from 2022 to 2024. The samples were collected from wild boars hunted during different hunting events on 21 game estates located in seven different provinces of mid-western Spain (Figure 1). The area predominantly features a continental Mediterranean climate, characterized by hot, dry summers and mild, moderately wet winters. Wild boars in the different hunting estates typically share their habitat with other ungulates such as red deer (*Cervus elaphus*), roe deer (*Capreolus capreolus*), fallow deer (*Dama dama*), and European mouflon (*Ovis orientalis musimon*). Only samples that had been continuously stored at −20°C were included in the study.

### 2.2. PCV4 Diagnosis

The DNA extraction from the samples was conducted using an isolation kit Nukex Mag RNA/DNA (Gerbion, Kornwestheim, Germany), following manufacturer instructions using KingFisher; Flex Purification System. Extracted DNA was initially tested by quantitative real-time polymerase chain reaction (qPCR) to evaluate the positivity of the samples.

Sequences of the PCV4 genome, based on NC_055580.1 sequence, targeted by all the PCR used in the present study were chemically synthesized at Genscript Biotech (Leiden, the Netherlands) including the region comprised 100 bp upstream and downstream of the primer location and cloned in a standard plasmid (pUC57), which was used as a positive control. Analytical sensitivity was assessed by performing serial dilution of the plasmid, allowing a detection of up to LoD of 11.1 copies/µL [23]. The specificity was assessed by testing several samples positive to relevant pathogen of swine interest, including PCV1-4. The method described by Zhang et al. [1], was implemented, setting the PCV4 final concentrations of 0.6 and 0.3 µM of primers (PCV4DF and PCV4DR) and probe (PCV4-prob), respectively. Afterward, a conventional PCR was performed on PCV4 positive samples in order to obtain amplicons for sequencing. Dedicated primers were designed with Primer3Plus [24]. All reactions were performed with the Platinum II Hot-Start PCR Master Mixes (Thermo Fisher Scientific, Waltham, MA, USA), in a Biometra TAdvanced Thermal Cycler (Analytik Jena GmbH, Jena, Germany). The final reaction settings included 1x Platinum II Hot-Start Green PCR Master Mix, 0.6 µM of each primer (PCV4_ORF2_F (5ʹ-TGAGGGAGGATGGGCAGTTGTATG-3′ and PCV4_ORF2_R 5ʹ-CACCACCCACAGATGCCAATCA-3′), 5 µL of extracted DNA and molecule grade water up to a final volume of 25 µL. The reaction conditions were 94°C for 2 min, followed by 45 cycles of 94°C for 15 s, 60°C for 15 s and 68°C for 30 s. Presence and specificity of the PCR amplicons were assessed through electrophoretic run on a SYBR Safe (Thermo Fisher Scientific, Waltham, MA, USA) stained agarose gel. All positive amplicons were purified using Applied Biosystems ExoSAP-IT PCR Product Cleanup Reagent (Thermo Fisher Scientific, Waltham, MA, USA). The same PCR primers were used for Sanger sequencing of the amplicons in both directions at Macrogen Europe (Milan, Italy).

### 2.3. Sequence and Phylogenetic Analysis

Chromatogram quality was assessed using FinchTV (2004–2006 Geospiza, Inc.), and consensus sequences were assembled using ChromasPro (Version 2.0.0, Technelysium Pty Ltd., South Brisbane, Australia). Complete ORF2 of PCV4 strains were retrieved from GenBank, with metadata on collection country, host, and date annotated in the sequence names when available. Poor-quality sequences, such as those containing premature stop codons, frameshift mutations, unknown bases, or apparent misalignments, as well as unverified entries, were excluded from the dataset.

All complete ORF2 sequences obtained were aligned at the amino acid level using the MAFFT [25] method implemented in TranslatorX [26] and subsequently back-translated to nucleotide sequences. Recombination events were analyzed in both datasets using the Genetic Algorithm for Recombination Detection (GARD) [27] implemented in Datamonkey [28]. Maximum likelihood (ML) phylogenetic trees were constructed using IQTree [29], selecting the substitution model with the lowest Bayesian information criterion (BIC).

### 2.4. Phylodynamic Analysis

The generated datasets were analyzed to estimate various population parameters, including the time to the most recent common ancestor (tMRCA), evolutionary rate, and viral population dynamics, using the Bayesian serial coalescent approach implemented in BEAST 1.10.4 [30]. For each subset, the nucleotide substitution model was selected based on the BIC score calculated with JmodelTest2 [31]. The molecular clock model was determined using marginal likelihood estimation through path-sampling and stepping-stone methods, as recommended by Baele et al. [32]. Viral population changes over time (relative genetic diversity: effective population size × generation time; Ne·τ) were reconstructed using the nonparametric Bayesian Skygrid model [33].

The spatial dispersal of PCV4 was inferred using a discrete state phylogeographic analysis as described by Lemey et al. [34]. An asymmetric migration model combined with Bayesian stochastic search variable selection (BSSVS) was employed.

A single run of 200 million generations was performed, and the results were analyzed using Tracer 1.7 [35]. Only runs with an estimated sample size (ESS) greater than 200 and adequate convergence and mixing after discarding the first 20% of data as burn-in were accepted. Parameter estimates were summarized as means with 95% highest posterior density (HPD) intervals. Maximum clade credibility (MCC) trees were constructed and annotated using TreeAnnotator (part of the BEAST package).

## 3. Results

### 3.1. Analysis of Retrospective Samples

A total of 302 samples of lymph nodes from wild boars were analyzed. From the total, 153 samples belonged to animals that were hunted between 2011 and 2015 and 149 samples belonged to animals that were hunted between 2022 and 2024. None of the animals showed overt clinical signs or macroscopic tissue lesion suggestive of viral infection, although chronic lesion suggestive of past bacterial of parasitic diseases were identified in some subjects. A total of 62 samples tested positive by qPCR, 57 positives belonged to the 2011–2015 sampling campaign and, five positives to 2022–2024. The estimated viral load, calculated from Ct values using the standard curve equation, was expressed as log_1_₀ (viral copies). The mean log_1_₀ viral load was 1.58, with a standard deviation of 1.16. Values ranged from a minimum of 0.04 to a maximum of 5.22. Afterward, 10 positives were obtained by conventional PCR, all of them belonged to 2011–2015, while no successful amplification was achieved for samples originating from the period 2022–2024 campaign using this PCR technique. The percentages of positivity by provinces were: 41.9% (18/43) in Badajoz, 7.2% (13/180) in Caceres, 57.1% (12/21) in Ciudad Real, 11.1% (1/9) in Cuenca, 78.9% (15/19) in Jaen, 14.3% (1/7) in Madrid, and 8.7% (2/23) in Toledo.

### 3.2. Sequence Analysis

A total of 10 complete ORF2 sequences were obtained (Acc. Numbers PV034664-PV034673) from strains collected in the Jaen, Ciudad Real, Badajoz, Toledo, and Cuenca provinces between 2011 and 2013. Including sequences previously obtained by Holgado-Martín et al. [7], a total of 13 Spanish sequences spanning the time period from 2011 to 2020 were available (Table S1). The mean genetic distance among these sequences was 0.69% (range: 0.00%–1.76%).

**Figure 2:**
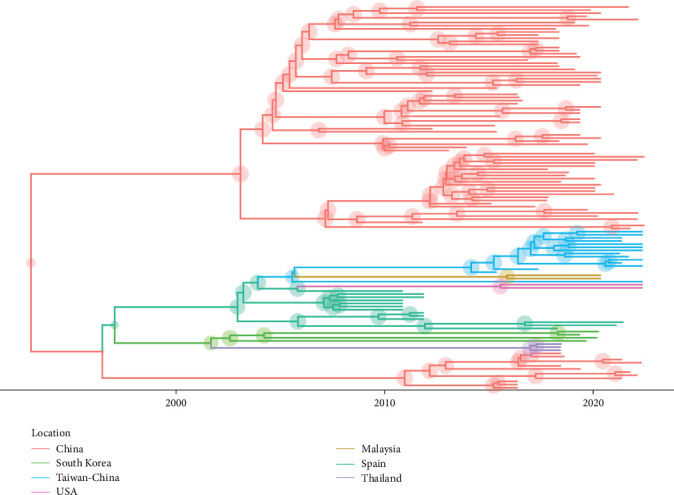
Maximum clade credibility tree of PCV4 strains based on ORF2 sequence. Countries where the virus ancestors were estimated to circulate are color-coded and the size of the corresponding node is proportional to the posterior support for the inferred location. The branch length is scaled in time (years).

Phylogenetic analysis, based on an international dataset, revealed the presence of two main clusters: one comprising exclusively Chinese sequences and another with a more diverse international profile, including sequences from Taiwan-China, Malaysia, South Korea, Thailand, the USA, and Spain (Figure 2). Although confidence in several nodes was low due to the limited genetic distance, Spanish strains formed a monophyletic clade, showing a closer relationship with one strain from the USA (RPP457621).

**Figure 3:**
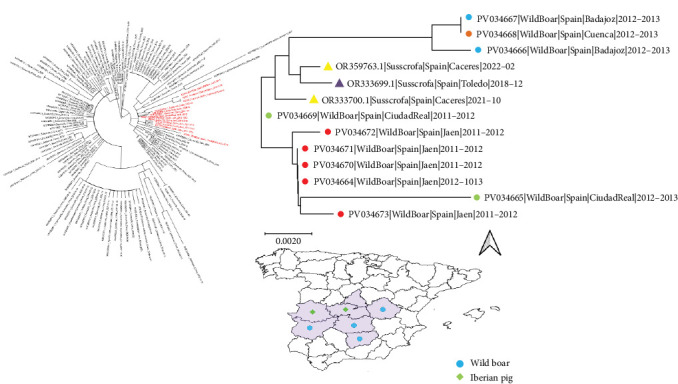
In the left panel, the maximum likelihood phylogenetic tree is based on the ORF2 of the strains included in the study, with Spanish strains highlighted in red. The upper right panel focuses on the phylogenetic relationships among Spanish strains, with different regions color-coded and host types represented by filled circles (wild boars) and triangles (domestic pigs). The lower panel provides a summary depiction of the Spanish provinces and the locations of positive samples, classified by host.

Within Spain, some geographical clustering was observed, with genetic distances reflecting the geographical proximity of provinces. For example, strains from Jaen formed a single cluster, which also included a single sequence from the neighboring province of Ciudad Real, sharing the northern border with this province. Similarly, another closely related strain originated from Ciudad Real, while progressively more genetically and geographically distant strains were from Cáceres and Toledo, located further to the northwest. Finally, the most distant clade included sequences from Badajoz and Cuenca, located to the west and east of Ciudad Real and Toledo, respectively. The sequences of the strains sampled from domestic rural pigs, obtained by Holgado-Martín et al. [7], were in an intermediate position from a phylogenetic point of view (Figure 3).

Phylodynamic analysis estimated the origin of PCV4 to approximately the mid-1990s (1993.69 [95% HPD: 1976.77–2008.89]) with an evolutionary rate of 6.76·10^–4^ (3.59·10^−4^−1.02·10^−3^). A steady and progressive increase in viral population size was reconstructed from the tMRCA until around 2020, when a slight decline was observed.

The phylogeographic analysis suggested that the Spanish strains originated from a single introduction event, estimated to have occurred around 2000. Of note, it was also inferred that the origin of both USA strains was linked to Spain (Figure 2). However, these findings should be interpreted with caution due to the limited availability of data, especially older sequences, which reduces the reliability of past movement inferences. Accordingly, while a Spanish origin of the international clade was estimated, the posterior probability was low, and South Korea was identified as a comparably likely source.

## 4. Discussion

PCV4 is the most recently identified porcine circovirus [36]. However, unlike PCV2 and PCV3, which have a worldwide distribution and were promptly reported in nearly all countries where their presence was investigated, the distribution of PCV4 appears to be much patchier. Initially identified in China, PCV4 was gradually reported in additional provinces within the country and later in other Asian countries, with several well-supported migration patterns described among them [37]. No evidence of extra-Asian circulation was available until recently when PCV4 was detected in Spain, Europe [7], and the United States [9]. This evidence, combined with the earliest detection of PCV4 DNA in tissues from 2012 and antibodies dating back to 2008 in China [13, 14], strongly suggests an Asian origin—estimated approximately in the mid-20th century—followed by local dispersal and only limited intercontinental spread.

The findings of the present study challenge this paradigm. Retrospective analysis of samples collected since 2011 confirms the presence of PCV4 in Europe for over a decade, likely even earlier based on the tMRCA estimation of the Spanish clade (~2000). Following its introduction into Spain, the virus appears to have persisted undetected, evolving over time in both wild boar and domestic rural pigs raised outdoor. The absence of clinical signs, as observed in the present study, likely contributed to the failure to detect the virus despite its prolonged circulation [38]. This may be attributable either to the low or absent virulence of the strain, or to the characteristics of the farming systems, where cofactors commonly involved in the emergence of clinical signs are often lacking. Unfortunately, the pathogenicity of PCV4 is still a matter of debate and no conclusive evidence can be obtained from the present study, Although no sequences from recently collected samples could be obtained in this study due to low viral titters, several samples collected between (2022–2024) tested positive, confirming ongoing viral circulation. A close genetic relationship was observed among strains sampled from wild boar and domestic pigs, supporting frequent strain exchange between these host populations. Such transmission events are highly plausible, considering the free-range farming system of Iberian pig breeds, which tested positive for PCV4. Similar evidence has been observed with PCV2 in Italy, particularly in Sardinia, where close contact between wild boar and free-range pigs facilitates effective viral transmission [39, 40].

The circulation of PCV4 limited to wild boar and rural pig breeds may also explain its geographical distribution and the observed relationship between genetic and geographic distance. This pattern suggests a progressive but slow spread from an initial introduction event mediated by animal movements, although the inference of the directionality of migration events is inappropriate given the limited data and sequence availability. The introduction of new pathogens has been previously linked to wild boar translocation from foreign countries [41]. However, in this case, the infected outdoor pigs occupied an intermediate position both phylogenetically and geographically, suggesting their potential role in virus spread, albeit within a limited area. On the other hand, a more widespread and long-distance spread would have been expected if significant circulation had occurred in intensively reared pigs.

The absence of PCV4 detection in the European intensive pig production system remains puzzling [23]. Limited contact opportunities due to high biosecurity or lower host susceptibility might explain this, but there is evidence against both hypotheses. Previous studies have demonstrated frequent viral exchange between wild and domestic extensive pig populations, including similar viruses like PCV2 and PCV3 [40, 42]. While the directionality of viral flow varies depending on the pathogen, this evidence reduces the likelihood of limited contact being the sole explanation. Alternatively, PCV4 might have a lower ability to replicate and spread in intensive farming systems. However, PCV4 has been detected in ~8% of pig samples in the U.S., where farming practices are comparable to those in Europe, and in intensive farms in Asia, which argues against host-related factors. A more nuanced scenario might involve a combination of host, environmental, and management factors, as well as competition with more virulent pathogens.

Interestingly, higher PCV3 prevalence has often been observed in wild and backyard pigs [43], and similar patterns have been noted for PCV2e in Italy [44], which circulates primarily in backyard pigs. Additionally, the previously thought-extinct PCV2c genotype has been found in specific ecological niches, such as feral pigs in Brazil and warthogs in Namibia [45, 46]. It is possible that PCV4, like PCV3 or certain PCV2 genotypes (e.g., PCV2c, PCV2e), may have lower fitness in intensive production systems due to a combination of host factors, environmental conditions, and pathogen competition.

Phylodynamic and phylogeographic analyses revealed the presence of two main clades: one consisting of Chinese strains and the other including sequences from multiple countries and continents. It is plausible that PCV4 was isolated in China for several decades due to limited animal trade and later spread to more internationally connected countries, leading to its progressive dispersion in Europe and the United States. However, the retrospective detection of PCV4 strains in Spain since 2011, combined with the tMRCA estimation of this clade around 2000—only slightly later than the updated overall PCV4 tMRCA estimation—raises questions about the actual viral origin. Alternative dispersal patterns cannot be excluded, and Spain may have played a role in the spread of PCV4 to the United States and other Asian countries. However, gaps between the estimated PCV4 origin and the oldest available sequences, as well as the limited data from other countries, hinder reliable inference of past viral movements. The long branches separating strains and clades likely conceal undetected connections, while the uneven sequence distribution may have biased the analysis. For example, the high number of Spanish sequences compared to other countries may have exaggerated the role of Spain in PCV4's origin and epidemiology. A similar bias could apply to the inferred Spanish origin of both US strains, which conflicts with phylogenetic analyses and may result from the parsimonious assumption of a single introduction event followed by local evolution. Overall, low posterior probabilities were associated with ancient location estimations, warranting caution in drawing any definitive conclusions in this regard.

Despite the findings of the present study challenge the previous hypothesis on PCV4 origin and history, recognizing the current uncertainties in PCV4's international distribution and epidemiology, further efforts are needed to improve knowledge of its global presence, including the use of archived samples in diagnostic activities. Such data could better reconstruct PCV4's history and dynamics, as demonstrated by the reduced uncertainty in tMRCA estimation achieved in this study compared to previous analyses. Similar approaches and perspectives could also be valuable for other emerging or neglected pathogens, whose evolutionary trajectories and epidemiological relevance may currently be underestimated due to limited surveillance and incomplete data.

Publishing negative results (i.e., studies where no positive samples were detected) is also of critical importance. Although often undervalued by researchers and scientific journals, these findings could help determine whether PCV4's patchy global distribution reflects true epidemiological patterns or simply the result of limited diagnostic and reporting efforts.

## Data Availability

All sequences produced in the study have been made available in GenBank.

## Supporting Information

Supporting Information Table S1. Summary table reporting the collection host, region, and date for the PCV4 strains sampled in Spain and included in the present study.

## References

[1] Zhang H.-H., Hu W.-Q., Li J.-Y. (2020). Novel Circovirus Species Identified in Farmed Pigs Designated as, *Porcine Circovirus*, 4, Hunan Province, China. *Transboundary and Emerging Diseases*.

[2] Segalés J., Sibila M. (2022). Revisiting Porcine Circovirus Disease Diagnostic Criteria in the Current Porcine Circovirus 2 Epidemiological Context.. *Veterinary sciences*.

[3] Saporiti V., Franzo G., Sibila M., Segalés J. (2021). Porcine Circovirus 3 (PCV-3) as a Causal Agent of Disease in Swine and a Proposal of PCV-3 Associated Disease Case Definition. *Transboundary and Emerging Diseases*.

[4] Segalés J., Allan G. M., Domingo M. (2005). Porcine Circovirus Diseases. *Animal Health Research Reviews*.

[5] Chen D., Zhang L., Xu S. (2023). Pathogenicity and Immune Modulation of Porcine Circovirus 3. *Frontiers in veterinary science*.

[6] Ha Z., Yu C., Xie C. (2021). Retrospective Surveillance of Porcine Circovirus 4 in Pigs in Inner Mongolia, China, from 2016 to 2018. *Archives of Virology*.

[7] Holgado-Martín R., Arnal J. L., Sibila M. (2023). First Detection of Porcine Circovirus 4 (PCV-4) in Europe. *Virology Journal*.

[8] Hung Y. F., Liu P.-C., Lin C.-H. (2024). Molecular Detection of Emerging *Porcine Circovirus* in Taiwan. *Heliyon*.

[9] Kroeger M., Vargas-Bermudez D. S., Jaime J. (2024). First Detection of PCV4 in Swine in the United States: Codetection With PCV2 and PCV3 and Direct Detection Within Tissues. *Scientific reports*.

[10] Nguyen V.-G., Do H.-Q., Huynh T.-M.-L., Park Y.-H., Park B.-K., Chung H.-C. (2022). Molecular-Based Detection, Genetic Characterization and Phylogenetic Analysis of Porcine Circovirus 4 From Korean Domestic Swine Farms. *Transboundary and Emerging Diseases*.

[11] Sirisereewan C., Nguyen T. C., Piewbang C., Jittimanee S., Kedkovid R., Thanawongnuwech R. (2023). Molecular Detection and Genetic Characterization of Porcine Circovirus 4 (PCV4) in Thailand During 2019–2020. *Scientific reports*.

[12] Tan C. Y., Thanawongnuwech R., Arshad S. S. (2023). First Molecular Detection of Porcine Circovirus Type 4 (PCV4) in Malaysia. *Tropical Biomedicine*.

[13] Ge M., Hu W., Ning K., Li S., Xiao C. (2021). The Seroprevalence of the Newly Identified Porcine Circovirus Type 4 in China Investigated by an Enzymed-Linked Immunosorbent Assay. *Transboundary and Emerging Diseases*.

[14] Hou C., Zhang L., Zhang Y. (2022). Phylogenetic Analysis of Porcine Circovirus 4 in Henan Province of China: A Retrospective Study From 2011 to 2021. *Transboundary and Emerging Diseases*.

[15] Li X., Chen S., Niu G. (2022). Porcine Circovirus Type 4 Strains Circulating in China are Relatively Stable and Have Higher Homology With Mink Circovirus Than Other Porcine Circovirus Types. *International Journal of Molecular Sciences*.

[16] Wang D., Mai J., Yang Y., Xiao C.-T., Wang N. (2022). Current Knowledge on Epidemiology and Evolution of Novel Porcine Circovirus 4. *Veterinary Research*.

[17] Xu T., Chen X.-M., Fu Y. (2022). Cross-Species Transmission of an Emerging Porcine Circovirus (PCV4): First Molecular Detection and Retrospective Investigation in Dairy Cows. *Veterinary Microbiology*.

[18] Xu T., Chen L., Huang B.-Z. (2023). The First Dog-Origin Porcine Circovirus Type 4 Complete Genomic Sequence Have High Homology With That of Pig-Derived Strains. *Frontiers in Microbiology*.

[19] Segalés J. (2012). Porcine Circovirus Type 2 (PCV2) Infections: Clinical Signs, Pathology and Laboratory Diagnosis. *Virus Research*.

[20] Sirisereewan C., Thanawongnuwech R., Kedkovid R. (2022). Current Understanding of the Pathogenesis of Porcine Circovirus 3. *Pathogens*.

[21] Malmsten A., Magnusson U., Ruiz-Fons F., González-Barrio D., Dalin A.-M. (2018). A Serologic Survey of Pathogens in Wild Boar (Sus Scrofa) in Sweden. *Journal of wildlife diseases*.

[22] Gortázar C., Ferroglio E., Höfle U., Frölich K., Vicente J. (2007). Diseases Shared Between Wildlife and Livestock: A European Perspective. *European Journal of Wildlife Research*.

[23] Franzo G., Ruiz A., Grassi L., Sibila M., Drigo M., Segalés J. (2020). Lack of Porcine Circovirus 4 Genome Detection in Pig Samples From Italy and Spain. *Pathogens*.

[24] Untergasser A., Cutcutache I., Koressaar T. (2012). Primer3—New Capabilities and Interfaces. *Nucleic Acids Research*.

[25] Katoh K., Standley D. M. (2013). MAFFT Multiple Sequence Alignment Software Version 7: Improvements in Performance and Usability. *Molecular Biology and Evolution*.

[26] Abascal F., Zardoya R., Telford M. J. (2010). TranslatorX: Multiple Alignment of Nucleotide Sequences Guided by Amino Acid Translations. *Nucleic Acids Research*.

[27] Kosakovsky Pond S. L., Posada D., Gravenor M. B., Woelk C. H., Frost S. D. W. (2006). GARD: A Genetic Algorithm for Recombination Detection. *Bioinformatics*.

[28] Weaver S., Shank S. D., Spielman S. J., Li M., Muse S. V., Kosakovsky Pond S. L. (2018). Datamonkey 2.0: A Modern Web Application for Characterizing Selective and Other Evolutionary Processes. *Molecular Biology and Evolution*.

[29] Trifinopoulos J., Nguyen L.-T., Haeseler A. V., Minh B. Q. (2016). W-IQ-Tree: A Fast Online Phylogenetic Tool for Maximum Likelihood Analysis. *Nucleic Acids Research*.

[30] Suchard M. A., Lemey P., Baele G., Ayres D. L., Drummond A. J., Rambaut A. (2018). Bayesian Phylogenetic and Phylodynamic Data Integration Using BEAST 1.10. *Virus Evolution*.

[31] Darriba D., Taboada G. L., Doallo R., Posada D. (2012). jModelTest 2: More Models, New Heuristics and Parallel Computing. *Nature Methods*.

[32] Baele G., Lemey P., Bedford T., Rambaut A., Suchard M. A., Alekseyenko A. V. (2012). Improving the Accuracy of Demographic and Molecular Clock Model Comparison While Accommodating Phylogenetic Uncertainty. *Molecular Biology and Evolution*.

[33] Hill V., Baele G. (2019). Bayesian Estimation of Past Population Dynamics in BEAST 1.10 Using the Skygrid Coalescent Model. *Molecular Biology and Evolution*.

[34] Lemey P., Rambaut A., Drummond A. J., Suchard M. A. (2009). Bayesian Phylogeography Finds Its Roots. *PloS Computational Biology*.

[35] Rambaut A., Drummond A. J., Xie D., Baele G., Suchard M. A. (2018). Posterior Summarization in Bayesian Phylogenetics Using Tracer 1.7. *Systematic Biology*.

[36] Opriessnig T., Karuppannan A. K., Castro A. M. M. G., Xiao C.-T. (2020). Porcine Circoviruses: Current Status, Knowledge Gaps and Challenges. *Virus Research*.

[37] Faustini G., Drigo M., Menandro M. L., Pasotto D., Giovanni F. (2022). Phylodynamic Analysis of Current, *Porcine Circovirus*, *4* Sequences: Does the Porcine Circoviruses Evolutionary History Repeat Itself?. *Transboundary and Emerging Diseases*.

[38] Holgado-Martín R., Risco D., Ramos A. (2025). Significant Detection of Porcine Circovirus 3 and Porcine Circovirus 4 in Wild Boars From Mid-Western Spain Without Apparent Sanitary Consequences. *Animals*.

[39] Dei Giudici S., Lo Presti A., Bonelli P. (2019). Phylogenetic Analysis of Porcine Circovirus Type 2 in Sardinia, Italy, Shows Genotype 2d Circulation Among Domestic Pigs and Wild Boars. *Infection Genetics and Evolution*.

[40] Faustini G., Poletto F., Baston R. (2024). D for Dominant: Porcine Circovirus 2d (PCV-2d) Prevalence Over Other Genotypes in Wild Boars and Higher Viral Flows From Domestic Pigs in Italy. *Frontiers in Microbiology*.

[41] Fernandez-de-Mera I. G., Gortazar C., Vicente J., Höfle U., Fierro Y. (2003). Wild Boar Helminths: Risks in Animal Translocations. *Veterinary Parasitology*.

[42] Franzo G., Faustini G., Legnardi M. (2023). Wilder Than Intense: Higher Frequency, Variability, and Viral Flows of Porcine Circovirus 3 in Wild Boars and Rural Farms Compared to Intensive Ones in Northern Italy. *Frontiers in Microbiology*.

[43] Franzo G., Grassi L., Tucciarone C. M. (2019). A Wild Circulation: High Presence of, *Porcine Circovirus*, 3 in Different Mammalian Wild Hosts and Ticks. *Transboundary and Emerging Diseases*.

[44] Faustini G., Tucciarone C. M., Legnardi M. (2023). Into the Backyard: Multiple Detections of PCV-2e in Rural Pig Farms of Northern Italy. *Preventive Veterinary Medicine*.

[45] Franzo G., Cortey M., Castro A. M. M. G. D. (2015). Genetic Characterisation of Porcine Circovirus Type 2 (PCV2) Strains From Feral Pigs in the Brazilian Pantanal: An Opportunity to Reconstruct the History of PCV2 Evolution. *Veterinary Microbiology*.

[46] Molini U., Franzo G., Gous L. (2021). Three Different Genotypes of Porcine Circovirus 2 (PCV-2) Identified in Pigs and Warthogs in Namibia. *Archives of Virology*.

